# Quantitative dynamic contrast-enhanced MR imaging can be used to predict the pathologic stages of oral tongue squamous cell carcinoma

**DOI:** 10.1186/s12880-020-00516-w

**Published:** 2020-10-16

**Authors:** Na Guo, Weike Zeng, Hong Deng, Huijun Hu, Ziliang Cheng, Zehong Yang, Shuqi Jiang, Xiaohui Duan, Jun Shen

**Affiliations:** 1 Department of Radiology, Sun Yat-Sen Memorial Hospital, Sun Yat-Sen University, No. 107 Yanjiang Road West, Guangzhou, 510120 Guangdong China; 2 Guangdong Provincial Key Laboratory of Malignant Tumour Epigenetics and Gene Regulation, Medical Research Centre, Sun Yat-Sen Memorial Hospital, Sun Yat-Sen University, Guangzhou, 510120 China; 3 Department of Nuclear Medicine, Peking University Third Hospital, No. 49 Huayuan Road North, Beijing, 100191 China

**Keywords:** Tongue neoplasms, Squamous cell carcinoma, Magnetic resonance imaging, Contrast media, Neoplasm staging

## Abstract

**Background:**

To investigate whether quantitative dynamic contrast-enhanced magnetic resonance imaging (DCE-MRI) pharmacokinetic parameters can be used to predict the pathologic stages of oral tongue squamous cell carcinoma (OTSCC).

**Methods:**

For this prospective study, DCE-MRI was performed in participants with OTSCC from May 2016 to June 2017. The pharmacokinetic parameters, including K^trans^, K_ep_, V_e_, and V_p_, were derived from DCE-MRI by utilizing a two-compartment extended Tofts model and a three-dimensional volume of interest. The postoperative pathologic stage was determined in each patient based on the 8th AJCC cancer staging manual. The quantitative DCE-MRI parameters were compared between stage I–II and stage III–IV lesions. Logistic regression analysis was used to determine independent predictors of tumor stages, followed by receiver operating characteristic (ROC) analysis to evaluate the predictive performance.

**Results:**

The mean K^trans^, K_ep_ and V_p_ values were significantly lower in stage III–IV lesions compared with stage I–II lesions (*p* = 0.013, 0.005 and 0.011, respectively). K_ep_ was an independent predictor for the advanced stages as determined by univariate and multivariate logistic analysis. ROC analysis showed that K_ep_ had the highest predictive capability, with a sensitivity of 64.3%, a specificity of 82.6%, a positive predictive value of 81.8%, a negative predictive value of 65.5%, and an accuracy of 72.5%.

**Conclusion:**

The quantitative DCE-MRI parameter K_ep_ can be used as a biomarker for predicting pathologic stages of OTSCC.

## Background

Oral tongue squamous cell carcinoma (OTSCC) is the most common malignancy of the oral cavity and comprises 25–40% of oral carcinomas [1]. It has a more aggressive clinical behavior and a relatively poor prognosis compared with other oral cavity and head and neck cancers [2]. However, the prognosis of OTSCC in early disease (TNM stage I–II) is better than that of advanced disease (TNM stage III–IV). For example, the five-year survival rate in patients with stage I disease exceeds 80% [3], but it drops below 40% for those with advanced disease at the time of diagnosis [4]. Likewise, the treatment selection is highly dependent on the TNM classification of staging at diagnosis. Early stage tumors may be treated using a single modality (surgery or radiotherapy), while advanced tumors frequently benefit from multimodality therapy. Thus, accurate staging of OTSCC prior to treatment is crucial for the treatment planning and prognosis prediction.

At present, the initial staging of OTSCC relies on a panel of procedures, including physical examination, direct endoscopic examination, computed tomography (CT), magnetic resonance imaging (MRI), and tumor tissue sampling. Among them, preoperative CT and MRI can help to assess tumor extension and infiltration as well as lymph node involvement. Notably, MRI is widely used to reveal the extent of soft tissue involvement and perivascular and perineural spread of OTSCC [5, 6]. However, there are limited quantitative imaging biomarkers with sufficient sensitivity or specificity to predict the prognosis or stage of OTSCC [6, 7]. Quantitative dynamic contrast-enhanced MRI (qDCE-MRI) can provide multiple pharmacokinetic parameters, such as model-free semiquantitative parameters and model-based quantitative parameters (parameters derived from pharmacokinetic model calculation) [6]. These parameters can fundamentally characterize the perfusion and vascularization of tissues and, indirectly, the state of the tumor [5–7]. Compared with semiquantitative parameters, quantitative parameters are less affected by wide variability in the MRI scanner, scanning sequence, temporal resolution, injection of contrast media, and image postprocessing calculation [5, 8]. Previously, the clinical stages of oral squamous cell carcinoma, including OTSCC, were found to be associated with quantitative parameters derived form DCE-MRI [9]. However, whether qDCE-MRI pharmacokinetic parameters can be used to predict the pathologic stages of OTSCC remains unknown so far.

In this study, DCE-MRI was prospectively performed in patients with OTSCC. The pharmacokinetic parameters were derived from DCE-MRI data by using a two-compartment extended Tofts model and three-dimensional volume of interest (3-D VOI). The purpose of this study was to determine the role of quantitative DCE-MRI in predicting the pathologic stages of OTSCC.

## Methods

### Patients

This prospective study was approved by the Ethics Committee of Sun Yat-Sen Memorial Hospital (Sun Yat-Sen University, Guangzhou, China), and written informed consent was obtained from all participants. Between May 2016 and June 2017, consecutive patients with suspected OTSCC on physical examination and/or CT were recruited. Patients were eligible for enrollment if they underwent surgical resection within 1 week after DCE-MRI examinations, and were pathologically diagnosed of OTSCC. Exclusion criteria included biopsy of tongue lesion before MRI examination, no surgical resection, previous history of chemotherapy or radiation therapy in the head and neck region, a lesion smaller than 1 cm in the maximum diameter (to avoid large partial volume effect during the measurement of qDCE-MRI parameters), obvious motion artifacts in the MRI images, contraindications to either gadolinium-based contrast material administration or MRI (e.g., metallic implant), or the inability to provide informed consent.

### MR imaging

All participants underwent DCE-MRI by using a 3.0 T scanner (Achieva, Philips Medical Systems, the Netherlands) with a 16-channel neurovascular coil (Philips Medical Systems, the Netherlands). MRI was performed within 1 week before the surgical procedure. The acquisition sequences included conventional multiplanar sequences MR imaging and DCE imaging. First, axial and sagittal T1-weighted imaging (T1WI) [repetition time (TR)/echo time (TE), 628/18 ms], axial T2-weighted imaging (T2WI) (TR/TE, 2643/90 ms) and coronal T2WI with fat suppression (TR/TE, 3000/60 ms) were obtained. The other main parameters included: flip angle, 90°; field of view [FOV], 220 × 220–230 × 230 mm^2^; section thickness/gap, 5.0/1.0 mm. Then, axial DCE-MRI was performed by using a 3-D T1-weighted high resolution isotropic volume examination (THRIVE) (TR/TE, 3.1/1.5 ms; flip angle, 12°; FOV, 230 × 230 mm^2^; slice thickness/gap, 8.0 mm/4.0 mm). The DCE acquisition included 110 phases with a temporal resolution of 3 s. Before DCE scan, variable flip angle images (2°, 4°, 7°, 9°, and 12°) were acquired for the calculation of T1 maps using the same sequence and parameters, except for the flip angle. After the initial two dynamic phases, a bolus injection of Gd-DTPA-BMA (Omniscan, GE Healthcare, Ireland) was administered at a dosage of 0.1 mmol/kg through the antecubital vein at a rate of 3 ml/s via a dual-head power injector (Spectris; Medrad, Pittsburgh, PA, USA), immediately followed by a 20 ml saline flush. The total duration of the DCE acquisition was 5.5 min. After DCE imaging, conventional axial, sagittal and coronal contrast-enhanced T1WI were obtained with the same parameters as the unenhanced T1WI.

### Imaging processing

The sequential DCE-MRI data were analyzed using a specialized quantitative analysis software (Omni Kinetics; GE Healthcare). A nonlinear registration framework utilizing the Free Form Deformation algorithm was firstly applied to correct the misalignment of body motion. Pharmacokinetic quantitative parameters were calculated from DCE images based on a patient-specific arterial input function (AIF, drawn on the common or external carotid artery ipsilateral to the tumor), the variable flip angle method and the two-compartment extended Tofts model [10]. To obtain the 3-D VOI, two experienced head and neck radiologists (N.G., with 4 years of experience in diagnostic imaging, and X.D. with 10 years of experience in diagnostic imaging), who were blinded for histologic results, independently drew the regions of interest (ROIs) slice by slice to encompass the entire lesion. Large feeding vessels and necrotic areas were excluded from the VOI. Quantitative parameters including K^trans^ (volume transfer constant), K_ep_ (reverse reflux rate constant), V_e_ (volume fraction of extravascular extracellular space), and V_p_ (volume fraction of plasma) were calculated. The pharmacokinetic parameters are described in Table 1.

**Table 1:** Quantitative DCE-MRI pharmacokinetic parameters

Parameters	Description of parameters	Units
K^trans^	Endothelial transfer constant	ml/min
K_ep_	Reflux rate of contrast agent from EES to plasma and equal to K^trans^/V_e_	ml/min
V_e_	Fractional EES volume (= K^trans^/K_ep_)	ml/ml
V_p_	Fractional plasma volume	ml/ml

*EES* extravascular extracellular space

### Surgery and histology

All patients were treated by surgery. Surgical resection was conducted within 7 days after MRI. The entire resected tongue specimens were processed for conventional histologic examination. The tumor invasion thickness, growth patterns (exophytic, ulcerated or endophytic), and pathologic TNM stages were recorded. The TNM staging was performed according to the 8th AJCC staging system [11].

### Statistical analysis

Statistical analyses were operated by using SPSS (Version 22.0, IBM SPSS Statistics, Armonk, NY, USA). All quantitative variables were showed as mean ± standard deviation. Interobserver agreement of the evaluation of qDCE-MRI parameters were evaluated by using the intra-class correlation coefficient (ICC). The ICC value closer to 1.00 represented better interobserver agreement. An ICC value greater than 0.75 indicates well to excellent agreement, while a value between 0.4 and 0.75 indicates fair to middle agreement. Data from the two readers were then averaged for analysis. The Shapiro–Wilk test was used to determine the normality of each qDCE-MRI parameter distribution. The numerical and categorical variables of clinicopathologic and qDCE-MRI parameters between stage I–II and stage III–IV lesions were compared using The Mann–Whitney U test and χ^2^ test, respectively. The association of each individual DCE-MRI parameter with tumor stage was determined by univariate binary logistic regression analysis. Significant parameters were chosen for multivariate binary logistic regression analysis to determine independent predictors of stages of OTSCC. The predictive efficiency of significant parameters was assessed by receiver operating characteristic (ROC) analysis. Furthermore, for each significant parameter, the area under the ROC curve (AUC), sensitivity, specificity, positive predictive value (PPV), negative predictive value (NPV) and accuracy were calculated. A two-sided *p* value < 0.05 was considered statistically significant.

## Results

### Study population

Of the 56 patients enrolled, 5 were excluded because of the maximum diameter of the lesion < 1.0 cm (*n* = 2) and the presence of obvious motion or metallic artifacts on MRI (*n* = 3). Finally, 51 patients were included in this study, including 31 male and 20 female patients aged from 23–90 years, with a mean of 55.5 ± 14.6 years. There was a total of 51 tongue tumors in the 51 patients. The clinicopathologic characteristics of these patients are shown in Table 2. The 51 patients were divided into the early stage group (stage I–II, *n* = 23) and the advanced stage group (stage III–IV,* n* = 28). The tumor thickness in stage III–IV lesions was greater than that in stage I–II lesions (*p* < 0.001). 78.3% (18/23) of stage I–II lesions had an exophytic or endophytic growth pattern, while 65.2% (15/23) of stage III–IV lesions showed an exophytic growth pattern (Table 2).

**Table 2:** The clinicopathologic characteristics of the patients (*n* = 51)

Characteristics	Stage I–II (*n* = 23)	Stage III–IV (*n* = 28)
Age (years)	50.8 ± 15.2 (23–86)	59.3 ± 13.1 (23–86)
Sex		
Female	11	9
Male	12	19
Tumor thickness (mm)	16.0 ± 6.1 (5–25)	20.1 ± 7.9 (6–36)
Growth pattern		
Exophytic	9	15
Ulcerated	5	7
Endophytic	9	6
TNM stage		
T stage		
1	10	1
2	13	10
3	0	7
4	0	10
N stage		
0	23	7
1	0	8
2	0	13
M stage		
0	23	28
1	0	0
Overall pathologic stage		
Stage I	10	0
Stage II	13	0
Stage III	0	9
Stage IV	0	19

### qDCE-MRI parameters

The ICC of qDCE-MRI parameters between the two readers was 0.878 for K^trans^ (95% CI 0.754–0.946), 0.863 for K_ep_ (95% CI 0.740–0.935), 0.902 for V_e_ (95% CI 0.786–0.962), and 0.922 for V_p_ (95% CI 0.810–0.974). Therefore, interreader agreement for the evaluation of the quantitative DCE-MRI parameters was good.

**Fig. 1:**
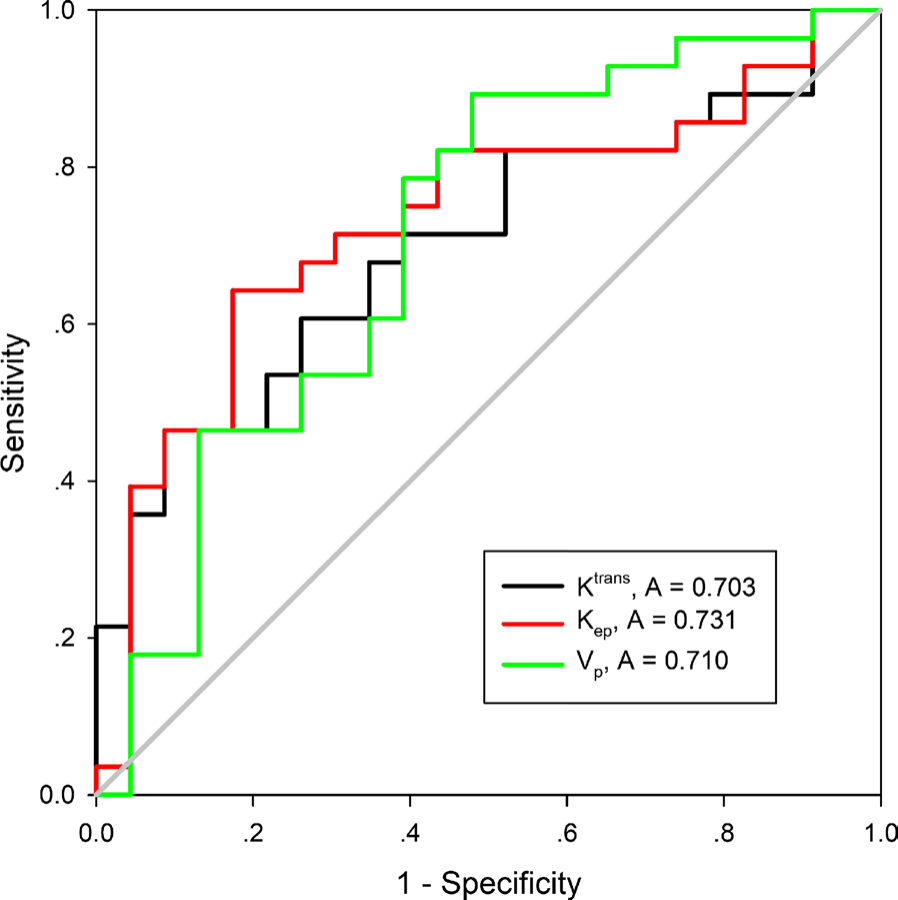
The ROC curves of K^trans^, K_ep_ and V_p_ for discriminating stage III–IV lesions from stage I–II lesions

**Fig. 2:**
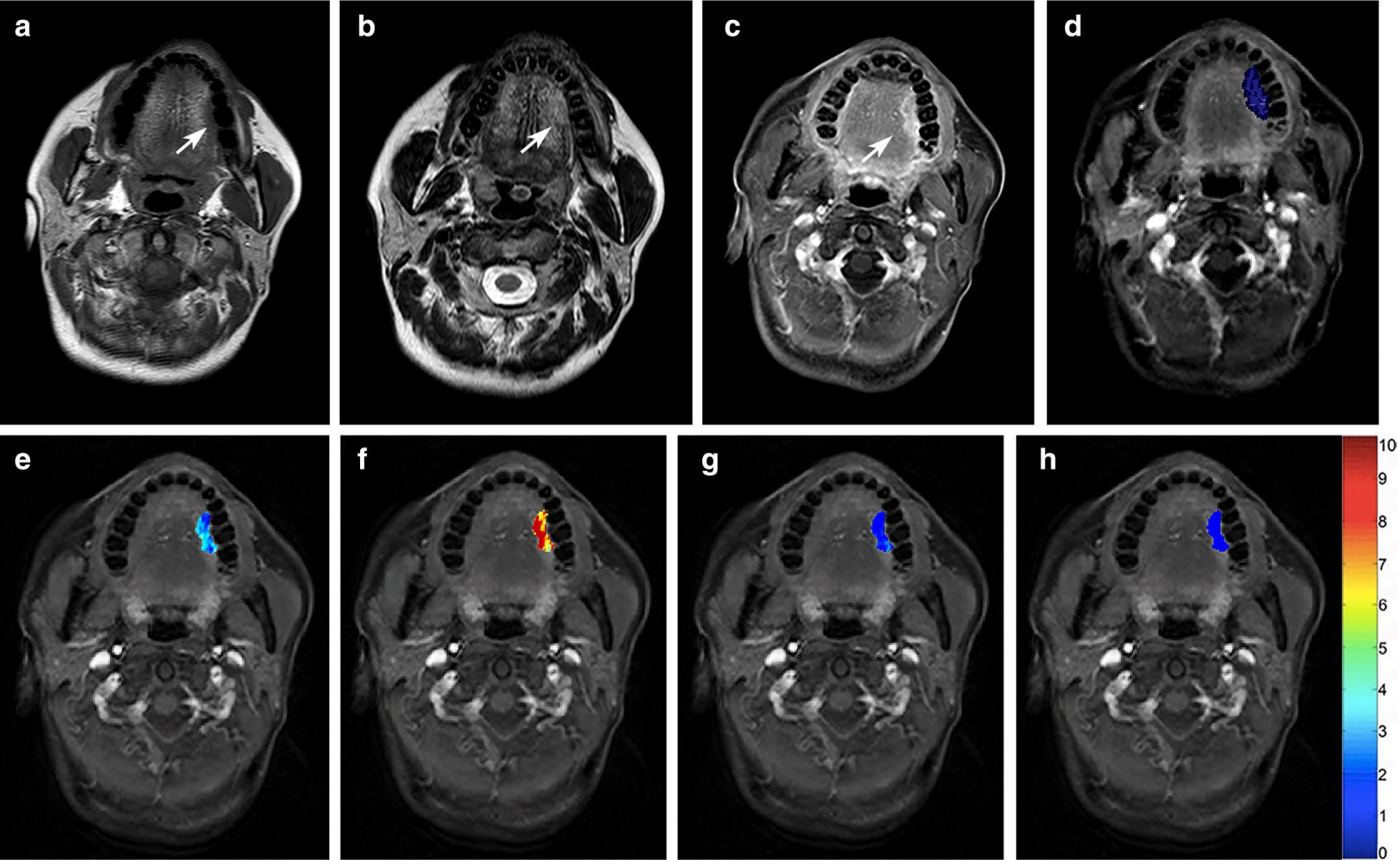
A well differentiated squamous cell carcinoma in the tongue, T1N0M0 (stage I). **a**–**c** The tumor was seen as a 20 mm × 12 mm × 11 mm mass (arrows) with isointense signal on T1WI (**a**) and hyperintense signal on T2WI (**b**), with heterogeneous enhancement on fat-suppressed contrast-enhanced T1WI (**c**). **d** DCE-MRI parameters are evaluated by a 3-D volume of interest (arrow). **e**–**h** Pseudocolorized maps show individual parameters derived from DCE-MRI. The measured K^trans^ (**e**) is 0.125 min^−1^, K_ep_ (**f**) is 0.995 min^−1^, V_e_ (**g**) is 0.127, and V_p_ (**h**) is 0.054

**Fig. 3:**
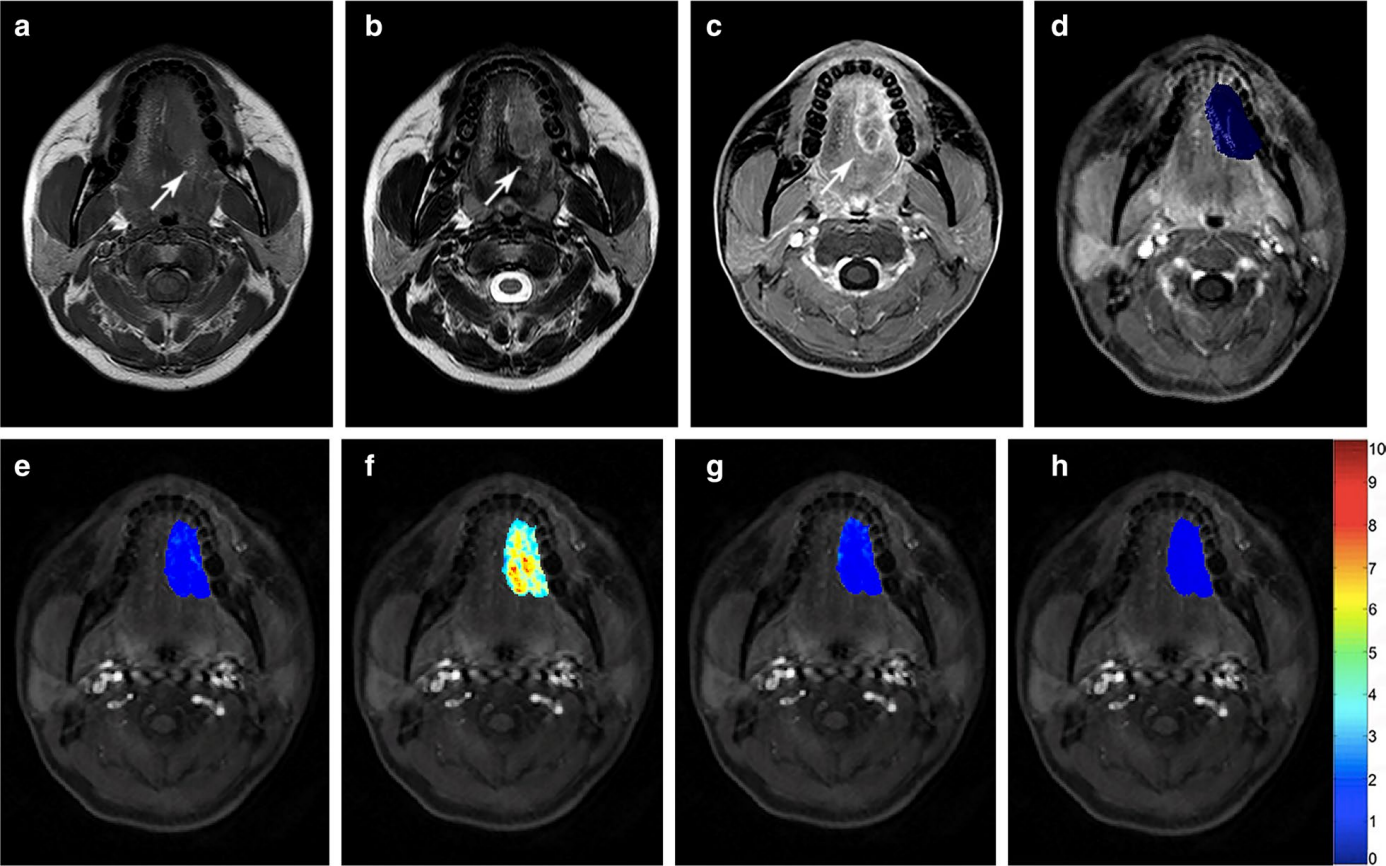
A well differentiated squamous cell carcinoma in the tongue, T2N2M0 (stage IV). **a**–**c** The tumor was seen as a 38 mm × 23 mm × 17 mm mass (arrows) with isointense signal on T1WI (**a**) and hyperintense signal on T2WI (**b**), with heterogeneous enhancement on fat-suppressed contrast-enhanced T1WI (**c**). **d** DCE-MRI parameters are evaluated by a 3-D VOI (arrow). **e**–**h** Pseudocolorized maps show individual parameters derived from DCE-MRI. The measured K^trans^ (**e**) is 0.043 min^−1^, K_ep_ (**f**) is 0.503 min^−1^, V_e_ (**g**) is 0.090, and V_p_ (**h**) is 0.009

The averages of the qDCE-MRI parameters and their comparison between stage I–II and stage III–IV lesions are shown in Table 3. The mean K^trans^, K_ep_ and V_p_ values of stage I–II lesions were significantly higher than those of stage III–IV lesion (*p* = 0.013, 0.005 and 0.011, respectively), while there was no significant difference in the V_e_ values (*p* = 0.325). Univariate logistic regression analysis showed that K^trans^, K_ep_ and V_p_ were associated with the tumor stage (*p* = 0.050, 0.025, and 0.039, respectively). ROCs of each significant parameter are shown in Fig. 1. The diagnostic performances of these parameters are shown in Table 4. Among the three significant parameters, K^trans^ had the highest NPV of 91.3%; K_ep_ had the highest AUC of 73.1%, the highest specificity of 82.6%, the highest PPV of 81.8% and the highest accuracy of 72.5%; and V_p_ had the highest sensitivity of 89.2% for discriminating stage III–IV lesions from stage I–II lesions. Two example cases are shown in Figs. 2 and 3. Further multivariate logistic regression analysis showed that K_ep_ was an independent predictor for stage III–IV lesions with an odds ratio (OR) of 0.035 (*p* = 0.025, Table 5).

**Table 3:** Quantitative DCE-MRI parameters for stage I–II and stage III–IV OTSCC lesions (*n* = 51)

Parameters	Stage I–II (*n* = 23)	Stage III–IV (*n* = 28)	*p *value*
K^trans^ (min^−1^)	0.149 ± 0.080	0.106 ± 0.057	0.013
K_ep_ (min^−1^)	0.806 ± 0.247	0.641 ± 0.221	0.005
V_e_	0.219 ± 0.140	0.188 ± 0.148	0.325
V_p_	0.017 ± 0.015	0.009 ± 0.008	0.011

*Mann–Whitney *U* test

**Table 4:** ROC analyses of qDCE-MRI parameters for discrimination between stage I–II and stage III–IV lesions

Parameters	AUC (95% CI)	Sensitivity(95% CI)	Specificity(95% CI)	PPV (95% CI)	NPV (95% CI)	Accuracy (95% CI)
K^trans^	0.703 (0.560–0.846)	0.867 (0.584–0.977)	0.583 (0.408–0.740)	0.464 (0.280–0.658)	0.913 (0.705–0.985)	0.647 (0.710–0.764)
K_ep_	0.731 (0.590–0.873)	0.643 (0.441–0.807)	0.826 (0.605–0.943)	0.818 (0.590–0.940)	0.655 (0.457–0.814)	0.725 (0.583–0.841)
V_p_	0.710 (0.562–0.857)	0.892 (0.706–0.972)	0.522 (0.311–0.726)	0.694 (0.517–0.831)	0.800 (0.514–0.947)	0.725 (0.583–0.841)

*AUC* area under the curve, *CI* confidential interval, *PPV* positive predictive value, *NPV* negative predictive value

**Table 5:** Multivariate logistic regression analysis of quantitative DCE-MRI parameters

Quantitative parameters	β value	*p* value	OR	95% CI
K^trans^	− 3.643	0.124	0.026	0.000–1.695 × 10^3^
K_ep_	− 3.356	0.025	0.035	0.002–0.661
V_p_	− 51.795	0.065	0.000	0.000–4.648 × 10^13^

*OR* odds ratio

## Discussion

Our study results showed that multiple pharmacokinetic parameters derived from qDCE-MRI were different between stage III–IV and stage I–II OTSCCs. Stage I–II lesions had higher K^trans^, K_ep_ and V_p_ values compared with stage III–IV lesions. K_ep_ was an independent predictor for stage III–IV lesions.

qDCE-MRI with tracer pharmacokinetic modeling has emerged as a versatile technique for characterizing the microvasculature function of the tumor. It can obtain the microvasculature function of tissue perfusion, vessel permeability and extracellular leakage space via monitoring the delivery and distribution of intravascular contrast agent [5, 6]. To date, qDCE-MRI has been widely used for tumor detection and characterization, therapy monitoring and predicting prognosis in various tumors such as protaste cancer, breast cancer, and gliomas [12]. Nevertheless, the relative low reliability of this technique restricts its adoption in routine clinical practice [5, 6]. There are many critical factors that influence the reliability of qDCE-MRI, including baseline T1 mapping, temporal resolution, and AIF in data acquisition [5, 6]. Baseline T1 mapping, which is used to compensate for the nonlinear relationship between MRI signal intensity and contrast agent concentration, is essential for accurate kinetic fitting of acquired DCE-MRI data [5]. In our study, we used five flip angles before injection of contrast agent to obtain the ideal baseline T1 mapping. Compared with other techniques of data acquisition for baseline T1 mapping (e.g., double flip angle technique, the inversion recovery technique, and the Look-Locker technique), the MFA method is now regarded as the technique of choice because it can provide more accurate, robust T1 mapping and kinetic parameter estimation with a short scan time but without sacrificing signal-to-noise ratio (SNR) [5, 6]. In addition, the temporal resolution of DCE-MRI in our study was 3 s, which was higher than what was found in most of the previous studies [9, 13, 14]. It has been suggested to use a temporal resolution from 1 to 5 s, after which the errors of quantitative DCE-MRI parameters calculation grow rapidly with the decrease in temporal resolution [15]. The chosen temporal resolution of 3 s in our study is an appropriate balance between the temporal resolution, SNR and spatial resolution, which allowed us to obtain high-quality DCE-MRI images and, in the meantime, capture the hemodynamic processes of contrast agents. AIF, which estimates the time course of the contrast agent concentration in the feeding arteries, is another crucial prerequisite for quantitative analysis of DCE-MRI. At present, the individual or population AIF can be used in DCE-MRI [5]. In our study, the AIF was extracted from individual patients rather than the population. Compared with the population AIF applied by previous studies [9, 13, 14], individual AIF could reflect the real AIF more closely, as it takes contrast agent injection rates and doses into account and presumes small intersubject variabilities [16]. In addition to the above key points in data acquisition, a 3-D VOI was used in our study. To date, most of previous studies have used a two-dimensional ROI (2-D ROI) derive the pharmacokinetic parameters from DCE-MRI for tumor assessment in head and neck cancer, while few studies have used a 3-D VOI for tumor analysis [9, 17]. Compared with 2-D ROI for tumor analysis, 3-D VOI can obtain the volumetric parameters and the heterogeneity data of the whole tumor, thus theoretically can more accurately describe the physiological characteristics of lesions [18].

DCE-MRI has been determined as a useful tool for diagnosis and differential diagnosis of benign and malignant tumors in head and neck, characterizing metastatic cervical lymph nodes, evaluating tumor cell proliferation and microvessel attenuation, predicting treatment response, evaluating treatment outcome and prognosis in head and neck cancers [19–21]. Previously, DCE-MRI has been found to be useful for differential diagnosis between benign and malignant tongue lesions [22], as well as between squamous cell carcinoma and undifferentiated carcinoma in head and neck [23]. Results indicated that the mean slope of the time-intensity curve (TIC) derived from DCE-MRI in malignant tongue tumors was steeper than that in benign lesions [22]. And the semi-quantitative parameter AUC at initial 90 s was the most accurate parameter to distinguish squamous cell carcinoma from undifferentiated carcinoma [23]. A recent study has shown that the value of K^trans^, V_e_ and initial AUC obtained from qDCE-MRI in metastatic cervical lymph nodes from SCC were higher than that in benign lymph nodes [24]. Nevertheless, there have been a limited number of studies that have utilized quantitative DCE-MRI for predicting the staging of SCC in the head and neck, and discrepancies exist in the previous reports. For example, Chikui et al. found that the clinical T stage of oral squamous cell carcinoma is negatively correlated with K^trans^ and the N stage showed a negative correlation with K^trans^ and V_p_ [9]. In contrast, Leifels et al. reported that the K_ep_ was higher in HNSCC cancers with N2-3 stages; however, no differences were observed in DCE-MRI parameters between T1-2 and T3-4 tumors [17]. In our study, K^trans^, K_ep_ and V_p_ were found to be lower in pathologic stage III–IV lesions than in stage I–II lesions; these results are similar to that of Chikui et al. [8] but different from that of Leifels et al. [17]. This discrepancy might be related to the different protocol of DCE-MRI scanning as well as different method of pharmacokinetic analysis. In our study, the MFA method for T1 measurement, individual AIF, higher temporal resolution and 3-D VOI were applied. These data acquisition and DCE-MRI data analysis methods made our results more reliable than the previous studies, which used the dual flip angle method, population AIF, and temporal resolution of 3.5 ms [9] and 6 ms [17]. Additionally, the pathologic TNM stage was used in our study, which is different from the study by Chikui et al. [9], which applied the clinical TNM stage. It is reasonable that our results are more favorable for reference in clinical practice.

In this study, the advanced stage OTSCCs had lower K^trans^, K_ep_ and V_p_ values than early stage ones. It has been shown that DCE-MRI parameters, such as K^trans^ and K_ep_, are negatively correlated with tumor hypoxia [6]. In addition, the more invasive oral squamous cell carcinoma had more highly hypoxic areas but less vessel density because of the gradual destruction of microvessels during tumor growth [25–27]. K^trans^ is positively coupled to blood flow, microvessel permeability and surface area, while K_ep_ represents microvessel permeability [7, 28]. Numerous studies have demonstrated that K^trans^ was negatively correlated with the fraction of hypoxic cells in tumors [29, 30], and there is a strong positive correlation between K_ep_ and microvessel density in HNSCCs [18]. Therefore, the highly hypoxic areas but with low microvessel density might be an explanation why advanced stages of OTSCC had lower K^trans^ and K_ep_ values in our study.

In our study, multivariate logistic analysis showed that K_ep_ was an independent predictor for advanced stage OTSCC. K_ep_ had the highest predictive capability, with a sensitivity of 64.3%, a specificity of 82.6%, a PPV of 81.8%, a NPV of 65.5%, and an accuracy of 72.5%. K_ep_ was more valuable for predicting the staging of OTSCC compared with other DCE-MRI parameters such as K^trans^ and V_p_. K_ep_, which represents the rate constant between the plasma and extracellular space, and is regarded as a marker that directly reflects microvessel permeability [18]. Previous studies have shown that the K_ep_ positively correlated with the mean blood vessel count and mean vessel area fraction parameter [18]. The advanced stage OTSCCs commonly had less vessel density because of the highly hypoxic areas [31], resulting in its lower K_ep_ within tumors. Taken together, our results indicated that K_ep_ can be used as a valuable predictive biomarker for tumor staging of OTSCC.

There was no significant difference in V_e_ between the stage III–IV lesions and the stage I–II lesions in our study. V_e_, which represents the volume of the extravascular extracellular leakage space, was mainly influenced by cellular density and tumor interstitium [8]. Pathologically, the cellular density and tumor interstitium of OTSCCs were variable among different stages and grades of tumor. Cell proliferation in advanced OTSCCs may be more intensive than that of early stage OTSCCs, which would cause high cellular density and contractible tumor interstitium resulting in low V_e_. Nonetheless, the rapidly growing late stage OTSCCs may have large regions suffering from chronic or acute hypoxia in the central area, which may lead to a focal or extensive apoptotic response and then decrease the cellular density [32, 33]. Therefore, V_e_ can be influenced by the varying cellular density between advanced and early stages of OTSCC; thus, it is less robust for predicting the stages of OTSCC compared with other qDCE-MRI parameters.

Our study had several limitations. First, the number of patients included was relatively small; a larger cohort is needed to confirm our results in a future investigation. Second, the enrolled patients did not receive follow-up. As a result, the correlation between qDCE-MRI parameters and survival outcomes remains unknown. Future follow-up investigation is needed to determine whether this method could be used to predict survival outcomes in patients with OTSCC.

## Conclusion

In summary, our study results showed that the mean K^trans^, K_ep_ and V_p_ values were higher in stage I–II OTSCCs than in stage III–IV OTSCCs. K_ep_ was an independent predictor of stage III–IV OTSCCs. The quantitative DCE-MRI-derived parameter K_ep_ can be used as a predictive biomarker for pathologic stages of OTSCC.

## Data Availability

The datasets used and analyzed during the current study are available from the corresponding author on reasonable request.

## Abbreviations

OTSCC: oral tongue squamous cell carcinoma
DCE-MRI: dynamic contrast-enhanced magnetic resonance imaging
THRIVE: T1-weighted high resolution isotropic volume examination
ROI: region of interest
3-D VOI: three-dimensional volume of interest
K^trans^: volume transfer constant
EES: extravascular extracellular space
K_ep_: reverse reflux rate constant
V_e_: volume fraction of EES
V_p_: volume fraction of plasma
ICC: intraclass correlation coefficient
ROC: receiver operating characteristic
AUC: area under the receiver operating curve
PPV: positive predictive value
NPV: negative predictive value
OR: odds ratio
CI: confidence interval
